# Novel insights into the neuroendocrine control of inflammation: the role of GR and PARP1

**DOI:** 10.1530/EC-13-0079

**Published:** 2013-12-19

**Authors:** Fernando Aprile-Garcia, María Antunica-Noguerol, Maia Ludmila Budziñski, Ana C Liberman, Eduardo Arzt

**Affiliations:** 1 Instituto de Investigación en Biomedicina de Buenos Aires – CONICET, Partner Institute of the Max Planck SocietyBuenos AiresArgentina; 2 Departamento de Fisiología, Biología Molecular y Celular, Facultad de Ciencias Exactas y NaturalesUniversidad de Buenos AiresBuenos AiresArgentina

**Keywords:** neuroendocrinology, inflammation, glucocorticoid receptor (GR), poly (ADP-ribose) polymerase 1 (PARP1)

## Abstract

Inflammatory responses are elicited after injury, involving release of inflammatory mediators that ultimately lead, at the molecular level, to the activation of specific transcription factors (TFs; mainly activator protein 1 and nuclear factor-κB). These TFs propagate inflammation by inducing the expression of cytokines and chemokines. The neuroendocrine system has a determinant role in the maintenance of homeostasis, to avoid exacerbated inflammatory responses. Glucocorticoids (GCs) are the key neuroendocrine regulators of the inflammatory response. In this study, we describe the molecular mechanisms involved in the interplay between inflammatory cytokines, the neuroendocrine axis and GCs necessary for the control of inflammation. Targeting and modulation of the glucocorticoid receptor (GR) and its activity is a common therapeutic strategy to reduce pathological signaling. Poly (ADP-ribose) polymerase 1 (PARP1) is an enzyme that catalyzes the addition of PAR on target proteins, a post-translational modification termed PARylation. PARP1 has a central role in transcriptional regulation of inflammatory mediators, both in neuroendocrine tumors and in CNS cells. It is also involved in modulation of several nuclear receptors. Therefore, PARP1 and GR share common inflammatory pathways with antagonic roles in the control of inflammatory processes, which are crucial for the effective maintenance of homeostasis.

## Introduction

The inflammatory response is a physiological process that protects the organism against infection and pathogens and repairs tissue damaged by injuries. It is normally beneficial to the organism, provoking the activation of various proinflammatory mediators in order to remove the damaging agent and restoring tissue function and structure (1). Biologically, inflammation advances through several stages. At the cellular level there is a marked response to proinflammatory stimuli, and as a result cytokine and chemokine cascades are initiated (2). The increase in these inflammatory mediators – cytokines, chemokines, growth factors, receptors, enzymes, and adhesion molecules – is considered pivotal for the progression and propagation of inflammation. On a molecular level, the appearance of proinflammatory signals culminates predominantly in the activation of activator protein 1 (AP1) and nuclear factor-κB (NF-κB). In turn, both transcription factors (TFs) induce the expression of the aforementioned inflammatory mediators, thus propagating cellular inflammation (3, 4, 5).

However, return to homeostasis – in which the neuroendocrine system has a paramount role – is necessary, considering that if the inflammatory process itself is prolonged it can lead to tissue injury and states of chronic inflammation and autoimmunity (6). This dysregulation has been identified as one of the major pathophysiological mechanisms underlying life-threatening human diseases (6, 7). Because of this determinant role in disease and also because inflammation is activated not only by infectious but also by environmental, behavioral, and psychological stimuli, inflammation is emerging as a main player controlling the balance between stress experience and human health (8). Indeed, there are mechanisms for the appropriate termination of the inflammatory response, and deficiencies in these mechanisms contribute to the appearance of inflammatory diseases.

After an injury, an important feature of the inflammatory response is the local release of a number of inflammatory mediators such as cytokines (interleukin 1 (IL1), IL6, and tumor necrosis factor α (TNFα)), which then act in the CNS activating the hypothalamic–pituitary–adrenal (HPA) axis, the main component of the endocrine stress response. IL1 and other cytokines act on the brain via several communication pathways: i) primary afferent neurons that innervate the periphery; ii) a humoral pathway that involves production of proinflammatory cytokines by macrophage-like cells and posterior diffusion across the blood–brain–barrier; and iii) cytokine receptors on endothelial cells of brain venules which mediate local production of prostaglandins (9, 10). This results in a neuroendocrine cascade of hormone signals that begins in the brain and ends with glucocorticoid (GC) secretion (cortisol in humans and corticosterone in rats, mice, and other species). When stimulated, neurons in the paraventricular nucleus of the hypothalamus release corticotropin-releasing hormone and arginine vasopressin. These factors cause secretion of adrenocorticotropic hormone in the anterior pituitary, which is released into the systemic circulation causing synthesis and secretion of GCs by the adrenal cortex (11, 12). The inflammatory response is mainly terminated by GCs, the end product of HPA axis activation, by a well-defined mechanism we describe below (13, 14, 15, 16, 17).

## Glucocorticoids

GCs are key neuroendocrine regulators of the inflammatory response. In a neuroendocrine-inflammatory feedback pathway, activation of the HPA axis leads to a rise in systemic GC levels which feedback and control the inflammatory response. Through this loop, GCs have an active participation in the interaction between the cellular components of the immune system and the neuroendocrine system, thus assuring maintenance of homeostasis avoiding excessive inflammatory effects that could be deleterious (10, 18). GCs are vital hormones that regulate a wide array of functions. Among others, they regulate metabolism, the immune response, neuronal survival, and neurogenesis, so also regulating behavioral function (11, 19). Thus, GCs are released in response to physical, emotional, and/or metabolic stress, and their effects serve as adaptive responses to stressful circumstances.

GCs belong to the steroid hormone family, a group of small lipophilic compounds derived from cholesterol, its common precursor. Steroid hormones are generally grouped according to the receptors they bind and their biological activity: progestins, androgens, estrogens, and corticoids. In turn, corticoids can be divided in mineralocorticoids, which regulate ion transport, and GCs, which have a wide variety of activities, including resistance to stress and immunosuppressive and anti-inflammatory actions (11, 19). Owing to their lipophilic nature, steroid hormones can freely diffuse through the cell membrane and bind to their cytoplasmic receptors. At the cellular level, the action of GCs is first regulated by activity of the enzyme 11β-hydroxysteroid dehydrogenase type 1 (11β-HSD1), which interconverts inactive GCs to their active counterparts, thus determining activation of GC before receptor binding. It has been reported that TNFα and IL1β increase the expression and activity of 11β-HSD1 in mesenchymal stromal cells, and combined treatment with GCs enhances this effect synergically (20, 21, 22). This further stimulation of 11β-HSD1 expression by GCs may be a mechanism to selectively increase local GC action during inflammation (23, 24). GCs exert their biological effects binding to the glucocorticoid receptor (GR), which is a ligand-activated TF that regulates the expression of target genes, either positively or negatively (18, 25). In an uninduced state, the GR resides predominantly in the cell cytoplasm in an inactivated form as part of a multimeric chaperone complex, consisting of several heat shock proteins and immunophilins. This complex keeps the ligand-binding pocket of the GR receptive to hormone binding and inactivates the nuclear localization signal (NLS). Once GCs bind to the GR, there is a conformational change in the receptor that allows the GR to dissociate from some components of the chaperone complex and expose the NLS, so the GR is able to move freely and translocate into the nucleus (26, 27). Consequently, ligand-bound GR gives rise to positive or negative transcriptional effects.

## Transcriptional regulation

Promoter activation of GR transcriptional targets can be elicited by different mechanisms: binding of dimeric, activating GR into GC response elements (GRE); DNA binding of the GR in a concerted manner with TFs; or binding of the GR to a TF by means of a tethering mechanism. The transactivation results in the expression of a number of anti-inflammatory proteins such as NF-κB inhibitor α (IκBα), GC-induced leucine zipper (GILZ), and dual-specificity phosphatase (DUSP) and IL10 (4, 28). However, the anti-inflammatory effects of GCs are mostly mediated via the interference elicited by a monomeric GR with the transactivation capacity of TFs, such as NF-κB and AP1, via a tethering mechanism named transrepression (18, 29, 30, 31). Also, GR can negatively regulate transcription by competing for an overlapping binding site (competitive GRE) or via DNA-binding with another TF (composite GRE), or else sequestering a DNA-bound TF (25). Thus, several TFs – NF-κB, AP1, Sp1, STAT3 among others – can work in concert with the GR regulating the fine-tuning of transcription, either in a positive or negative manner (32). The most prominent anti-inflammatory effects of GCs are elicited mainly by inhibiting the activity of TFs such as AP1 and NF-κB, which are involved in the activation of proinflammatory and immunoregulatory genes such as inflammatory cytokines (e.g. IL1β, IL6, and TNFα), cytokine receptors, adhesion molecules (e.g. ICAM1, VCAM, and E-selectin), and chemotactic proteins and thus are indispensable for the propagation of inflammation (29, 33). All of these genes have one or more NF-κB and/or AP1-responsive elements in their promoters (18, 29, 34). Indeed, the first described anti-inflammatory activity of GCs involving transrepression was the physical interaction between GR and AP1 (35), which results in the inhibition of inflammatory cytokine IL2 expression (36). NF-κB regulates a wide array of inflammatory cytokines, such as TNFα and IL1β. Thus, inhibition of NF-κB activity mediated by GCs is a main feature of the GR-elicited anti-inflammatory action (4, 31, 37). It also inhibits NFAT-dependent IL2 transcription, by a mechanism involving the cooperative binding between NFAT and AP1 dimers by protein–protein interaction (38). The main mechanism of the GR action over these TFs is via the transrepression mechanism: the activated GR tethers to the TF, modulating transrepression of the targeted genes, thereby inhibiting gene expression. The GR does not inhibit the binding of NF-κB or AP1 to their responsive elements in the gene promoter. Instead, GR binds proximal to the NF-κB or AP1-binding site and interacts with these TFs: for example, interaction of the GR with the C-terminal activation domains of NF-κB p65 is determinant for its repressive effect on NF-κB-regulated gene expression (39). The cross-talk mechanism is not restricted to these well known TFs, but has been expanded in the past years to other factors including CREB, NFAT, STAT, T-bet, and GATA-3 (40, 41, 42).

## GCs anti-inflammatory effects and therapeutic applications

The interplay mentioned between cytokines, HPA axis activation and GCs modulation has an important role in the control of inflammation, given the fact that the increase in GCs levels elicited after HPA-axis activation by proinflammatory cytokines contributes to maintain homeostasis during immune response (43). A situation of an excessive tissue inflammation plays a critical role in the development of chronic inflammatory disorders. The administration of GC analogs is often employed in the clinic in situations of unresolved inflammatory processes, representing the first line of drugs used to help control the homeostasis of organism in allergic, inflammatory, and autoimmune disorders (44, 45, 46). It is generally accepted that the transrepression mechanisms mediated by the GR sustain the beneficial anti-inflammatory action of GCs, whereas their side effects are due to direct binding of GR to responsive promoter elements as depicted before. Along with this notion, the ideal GC analogs for therapeutic purposes should be those that have only high transrepression but very low residual transactivation properties, therefore, causing minimal side effects. Several steroidal and nonsteroidal ligands of GR have been reported to have this dissociated function between transactivation and transrepressive mechanisms (44, 45, 46, 47). Thus, these compounds repress activity of not only NF-κB and AP1 but also other TFs, showing anti-inflammatory and immunosupressive activities *in vivo* (48, 49, 50, 51). However, GCs can induce gene transcription not only by binding GRE elements but also in combination with other TFs and via promoter elements that do not involve GR dimerization or DNA interaction; therefore, unexpected secondary side effects might appear (52). Consequently, the future search for GR ligands should balance between undesirable transactivation and efficient transrepressive properties *in vivo* (44, 46). Considering the high percentage of GCs resistance seen daily in the clinical practice, it would be important to know whether these selective GR modulators are more efficient than traditional GCs to overcome resistance minimizing side effects (53).

## Hormone receptor modulation for the control of inflammation

Members of the family of steroid hormones, as is the case for GCs, have a big influence on a wide variety of physiological responses, leading to homeostasis, including maintenance of neuroendocrine circuits, both in health and disease. These effects are mediated by specific receptor activation. Steroid receptors are members of the nuclear receptor (NR) superfamily. They can be grouped into four classes according to their ligand-binding, DNA-binding, and dimerization properties: steroid receptors – progesterone receptor (PR), androgen receptor (AR), estrogen receptor (ER), mineralocorticoid receptor, and GR – RXR heterodimers – including retinoic acid receptor (RAR) and thyroid hormone receptor – and orphan receptors (54). As previously detailed for the GR, the other members of the NR superfamily also contribute both positively and negatively to gene expression after a stimulus, as well as interacting and interfering with other signaling pathways (e.g. inhibition of gene activation by NF-κB or AP1), thus representing an important regulatory link between the endocrine and immune system (34). Dysregulation of these processes can lead to disease. As such, dysregulation of GR, as well as other NRs, have consequences in the control of inflammation. The functional interaction between NRs and NF-κB has been proposed to play a role in tumorigenesis *in vivo* (55, 56). Over the past few years, an increasing body of evidence reveals that NF-κB plays a critical role in tumor development. The potential of NRs to modulate the activity of this widespread TF has been reported and their therapeutic potential has been illustrated (34, 57).

Treatments targeting each hormone receptor are generally employed to reduce pathological signaling through these receptors thereby to inhibit malignant cell proliferation. Although these treatments are effective for many patients, resistance is also a common feature of these therapies (58). Thus, new treatment strategies are needed in these cases. Specific intracellular modulation of receptor activity may be one feasible alternative. In this regard, NRs are known to be modulated by different mechanisms and molecules, involving regulation of its expression, post-translational modifications, and activity modulation by coregulators (34, 59, 60, 61, 62).

In this matter, one specific molecule that has caught the attention of researchers in the last few years is poly (ADP-ribose) polymerase 1 (PARP1). This long-known protein is starting to reveal new and exciting functions, some of them related with endocrine pathologies, by means of interaction and modulation of NRs activity.

## PARP: introduction and transcriptional regulation

PARP conform a family of 18 proteins that were identified by homology searching and characterization *in silico* (63, 64). Members of this family share a highly conserved PARP signature motif in the catalytic domain. These enzymes catalyze the addition of PAR on target proteins. PAR is a large and negatively charged polymer that works as a post-translational modification. The cellular content of PAR is produced by PARP's catalytic activity, which polymerizes ADP-ribose units from donor NAD+ molecules on target proteins (65, 66). This modification most likely occurs on glutamate, aspartate, and lysine residues. There has been some progress on elucidating the specific sites of PAR addition (67). The covalent PAR attachment alters the activity of the modified proteins by means of charge and steric effects, thus altering protein–protein interactions, nucleic acid–protein interactions, enzymatic activity, and subcellular localization (68). The most studied member of the family is PARP1, a nuclear enzyme with a wide variety of functions. It was originally described as capable of binding to damaged DNA and thus become activated, and was therefore described as an important mediator of the responses to DNA damage (69). Over the last decade, it has been shown that PARP1 not only mediates DNA repair, but it also has important roles in different nuclear processes such as replication, chromatin remodeling, transcription, and maintenance of genomic stability (70). The number of proteins known to be targets of PARP1 enzymatic activity is on permanent growth. It has been shown that PARP1 modifies histones, TFs, nuclear enzymes, and nuclear structural proteins. PARP1 parylates histones, thereby regulating chromatin structure (71). It also parylates a number of DNA repair proteins such as p53 (72). PARP1 has also been reported to parylate and alter the function of numerous TFs, including AP1, NF-κB, CTCF, and YY1 (73). Thus, the cellular functions of PARP1 are ultimately defined by protein parylation.

However, functions of PARP1 are not only mediated by its intrinsic activity of parylation but also due to association with different proteins, such as transcription-related factors (73). In particular, the role of PARP1 in gene regulation has received considerable attention (73, 74, 75), and it has been established that it can modulate gene expression under basal, signal-activated, and stress-activated conditions at different levels: i) modulating chromatin structure, ii) serving as a coregulator with DNA-binding TFs, and iii) modulating DNA methylation (70).

### Modulation of chromatin

The first reported effects of PARP1 on the genome were chromatin structure modulation and parylation of histones (76, 77) and were afterwards validated (78, 79). PARP1 binds to nucleosomes and interacts dynamically with different types of chromatin domains, thereby modulating chromatin structure (71). Activation of PARP1 promotes chromatin decondenzation and restoration of transcription (78). PARP1 localizes to the promoters of almost all actively transcribed genes (80), suggesting a role in promoting the formation of chromatin structures that are permissive to transcription (78, 80, 81).

### Transcriptional coregulation

Regulation of gene expression by PARP1 may also be accomplished by serving as a coregulator, acting together with the transcription machinery, other coregulators with enzymatic activities, and with sequence-specific DNA-binding TFs, such as NF-κB, Elk1, NFAT, Oct1, and Sox2. Interestingly, PARP1 can interact with NRs such as ER, PR, and RAR (65, 71, 73). The effect of PARP1 over these activators may be stimulatory or inhibitory and may require or not its enzymatic activity. PARP1 is enriched around the transcription start sites of the genes that are actively expressed, therefore is an excellent marker of active promoters. Remarkably, PARP1 was previously identified as the basal TFIIC (82) that coregulates RNA polymerase II preinitiation complex formation before TFIID binding, therefore enhancing gene transcription. Also, several reports have shown that PARP1 is responsible for assembling coregulator complexes at the promoter of target genes, functioning as a scaffold protein, without binding to DNA or requiring its catalytic activity, promoting the recruitment of other coregulatory enzymes required for transcription (70). For example, in response to proinflammatory stimuli, PARP1 facilitates direct physical interaction and functional cooperation between the acetyltransferase p300/CBP, the p50 subunit of NF-κB and the mediator complex (83, 84). In other cases, PARP1 has been described as a promoter-specific ‘exchange factor’, releasing inhibitory factors and recruiting stimulatory factors to TFs bound to these promoters (81, 85).

### Modulation of DNA methylation

It has been shown that PARP1 can affect the methylation of genomic DNA (86, 87). PARP1 regulates both the expression and activity of the DNA methyltransferase, Dnmt1 (88), and it was also described to directly interact with Dnmt1 after attachment of new PAR polymers, inhibiting Dnmt1 DNA methyltransferase activity (89).

## PARP1 and neuroendocrine mediators

**Figure 1:**
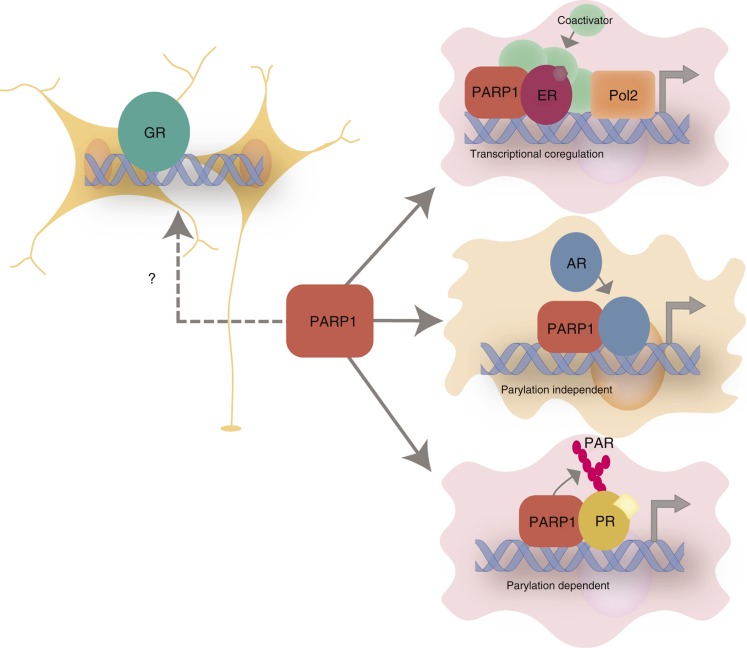
PARP1 regulation of nuclear receptors (NRs) in endocrine tissues. PARP1 regulates NRs transcriptional activity through different mechanisms depending on cell context. PARP1 induces the transcriptional activity of ligand-activated ER in the breast cancer cells by recluting transcriptional coactivators to ER target genes. PARP1 modulates AR–chromatin interaction in prostate cancer cells, thereby increasing AR-mediated transcription, in a parylation-independent manner. PARP1 induces ligand-activated PR-mediated transcription in breast cancer cells in a parylation-dependent manner. The effect of PARP1 on GR-mediated transcription in the neuroendocrine system has yet to be addressed.

PARP1 has been linked with the regulation of the activity of several NRs, specially in the modulation of endocrine processes. Particularly, it has been shown that PARP1 is involved in several NRs-mediated transcription (Fig. 1). PARP1 acts as a coregulator in the concert of a wide variety of transcriptional regulators that give temporal and spatial specificity to gene expression.

PARP1 has been described to be recruited to chromatin areas surrounding the estrogen response element present in the *pS2* promoter in 17β-estradiol (E_2_)-treated MCF7 cells as part of a specific coactivator complex recruited to the liganded ERα (81). In this regard, a rapid increase in PARP1 recruitment together with coactivators and Pol2 and the elimination of corepressors in response to E_2_ was reported, events that were necessary for transcriptional activation. Furthermore, pharmacological or genetic inhibition of PARP1 blocked ERα-dependent gene expression (81).

Another study (85) focused on PARP1 effects over RAR-dependent transcription. This study demonstrated a functional and physical interaction between PARP1 and RAR leading to RAR-mediated transcriptional activation, thus concluding that PARP1 is an essential coregulator for RA-induced gene expression *in vivo*. More specifically, PARP1 is a cofactor that makes the switch from inactive to active RAR-dependent promoters. This switch is determinant for the transcriptional status and constitutes an additional mechanism for gene regulation.

PARP1 coregulation of NRs activity has been shown to have a role on cancer growth and progression of endocrine tumors. In this line, PARP1 is involved in prostate and breast cancer, by means of modulating AR and PR respectively. In a recent report (90), it was shown that PARP1 has protumorigenic effects on positive-AR prostate cancer cells. PARP1 seems to be recruited to AR-dependent promoters, where it promotes AR occupancy and transcriptional function, by modulating AR–chromatin interaction. PARP1 inhibition reduced prostate-specific AR target genes. It is important to note that PARP1 regulation of AR activity is not attributable to parylation. There also seems to be a correlation between prostate cancer progression and PARP1 enzymatic activity, because this activity is enhanced on advanced prostate cancers. Furthermore, PARP1 activity is required for tumor cell growth *in vivo* and its targeting potently suppresses tumor cell proliferation, suggesting that PARP1 can be targeted on human prostate cancer to suppress tumor growth (90).

PARP1 also has a role in breast cancer, mediated by its interaction with the PR. It was first discovered that PARP1 was part of a protein complex that could interact *in vitro* with ligand-activated PR and assist on DNA binding (91). When the effects of PARP1 over the PR were evaluated in breast cancer cells treated with progestin, there was an enhanced PARP1 enzymatic activity (92). PARP1 activation also led to a global increase in PAR levels, essential for the modulation of the majority of progesterone-regulated genes. Inhibition of PARP1 blocked the downstream activation or repression of 85% of progestin target genes. As a consequence, given the multiplicity of genes affected, PARP1 could be a potential target for the pharmacological management of breast cancer. Along this line, new therapeutic approaches targeting breast cancer which involve PARP1 have been proposed (93, 94).

As mentioned, PARP1 interacts and regulates multiple NRs involved in endocrine maintenance. Interestingly, the putative interaction of PARP1 with the GR has not been explored yet. This interaction could be relevant in the maintenance of neuroendocrine circuits as PARP1 could be modulating the effects of the GR (Fig. 1). The review highlights that PARP1 is important in the inflammatory response, hence the coregulation with the GR might be relevant for their function.

## PARP1 in inflammation

As described previously for the GR, immune and inflammatory responses are the best-characterized PARP1-dependent biological responses (95). PARP1 is heavily automodified upon bacterial infection (96) and *Parp1*^−/−^ mice have proven to be resistant to inflammation in different experimental models, such as LPS-induced septic shock and streptozotocin-induced diabetes (97, 98). Interestingly, PARP-dependent proinflammatory responses are not limited to cells of the immune system: PARP is implicated in the pathological proinflammatory responses to stress in cells of the CNS as well. In contrast to the well characterized GR anti-inflammatory action, PARP1 activation in glial cells mediates the function of TFs that control the expression of genes of the inflammatory response, such as NF-κB and AP1. In models of cerebral ischemia, expression of genes such as *IL6*, *IL1B*, *COX2*, *iNOS*, and *ICAM1* is elevated, while in PARP1 knockout mice or after the administration of PARP inhibitors, expression of these genes is significantly reduced (99, 100, 101, 102, 103).

These findings led to the notion that PARP1 is an important mediator of inflammatory responses on cells subjected to different stimuli. In this aspect, it was already recognized almost two decades ago that PARP1 inhibitors have anti-inflammatory properties (104), being this a subject of still intense research. A considerable number of TFs known to be involved in the regulation of expression of inflammatory mediators have been shown to interact with PARP1. The first one to be identified was NF-κB (105, 106). Upon PARP1 deletion, gene expression induced by NF-κB was abolished, thus reducing proinflammatory cytokines (TNFα and iNOS) expression after LPS injury (106). These effects were also observed in the CNS. *PARP1*^−/−^ glial cells showed a diminished DNA-binding activity of NF-κB, with the subsequent reduction in expression of proinflammatory mediators including IL6, IL1β, TNFα, COX2, and iNOS (100). Afterwards, other TFs and cofactors that are involved in the regulation of inflammation were found to be modulated by PARP1, such as AP1 (97, 107), NFAT (108, 109), SIRT1 (110), and Sp1 (100). The precise mechanism of regulation of these TFs is still a matter of intense research, being a common point the fact that PARP1 activity enhances DNA-binding capacities of TFs. By regulating their activity, PARP1 ultimately regulates the expression of inflammatory cytokines such as TNFα, IL1β, IL6, and IL12, which in turn activate the expression of other cytokines, chemokines, iNOS, and COX2, suggesting that PARP1 plays an important role in several pathophysiological inflammatory responses.

As described earlier, PARP1 regulates transcription in a wide array of systems, including immune cells, endocrine tumors, and glial cells. As such, PARP1 involvement in neuronal and glial physiology is proving to be quite important. The relevance of PARP1 in the CNS is receiving considerable attention. PARP1 has been shown to be involved in different injury mechanisms affecting neurons. As previously described for GC-mediated apoptosis (30), it is already recognized that PARP1-mediated cell death is one of the dominant cell death process in many disease settings (111). PARP1 activation has been detected in various neurodegenerative disorders (112), with a role also identified for the GR in these pathologies (113, 114). It has been shown that elevated PARP1 activation levels are sufficient for neuronal death (115) and astrocyte death (116). In more chronic CNS disease, such as experimental autoimmune encephalomyelitis (EAE) model where there is an important inflammatory component, PAR accumulation has been found not only in astrocytes surrounding demyelinated EAE plaques but also to a lesser extent in microglia, oligodendrocytes, and neurons (117). Finally, autopsy samples from Alzheimer patients showed PAR accumulation in cortical pyramidal neurons and in astrocytes, suggesting PARP1 activation, with no PAR accumulation in microglia (118). PARP1 activation drives neuronal death elicited by fragments of peptide β-amiloid, implicating PARP1 in the pathogenesis of Alzheimer's disease (119). Astrocytic PARP activation seems to be quite a common feature of chronic neurodegenerative disorders, suggesting a key role for PARP1 in these inflammatory diseases.

**Figure 2:**
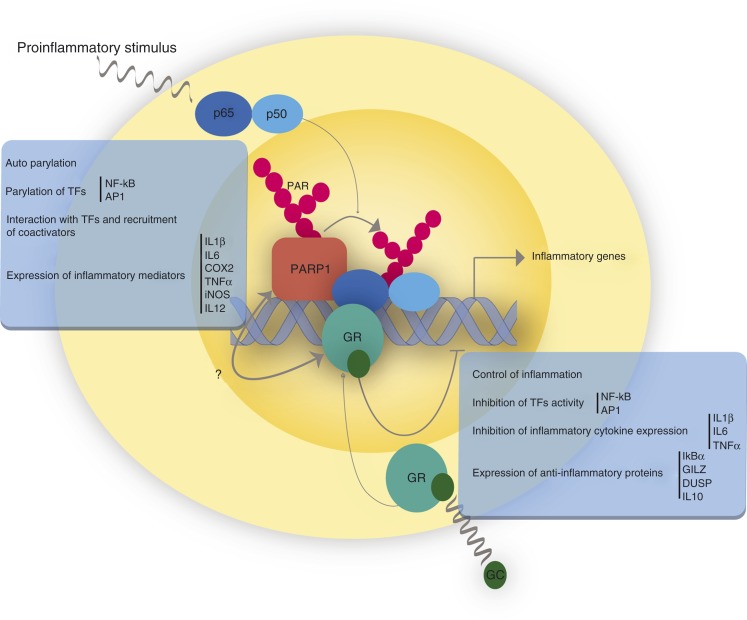
GR and PARP1 in inflammation. GR and PARP1 regulate inflammatory responses. GR inhibits the expression of inflammatory mediators through the modulation of the transcriptional activity of inflammatory transcription factors and expression of anti-inflammatory genes. On the contrary, PARP1 induces the expression of inflammatory mediators through stimulation of the transcriptional activity of inflammatory transcription factors. The interplay between GR and PARP1 in the final outcome of inflammatory responses remains to be elucidated.

Taking into account the data reviewed so far, both PARP1 and GR share common pathways. To explore the putative interaction between these two molecules, one interesting pathway to explore would be their opposing role in the transcription of proinflammatory cytokines, by means of antagonically regulating TFs activity such as NF-κB and AP1 (Fig. 2).

For example, interaction between PARP1 and GR may be involved in anti-inflammatory mechanisms driven by the GR. Upon ligand binding and traslocation to the nucleus, GR may reduce inflammatory effects mediated by PARP1 on NF-κB. One feasible mechanism for this could be that GR interaction with PARP1 reduces its activity on NF-κB or that GR competes with PARP1 for NF-κB binding. This last alternative is rather appealing, since it would provide a fast fine-tuning for NF-κB-mediated transcriptional regulation of inflammatory cytokines. Another possibility is that PARP1 may be modulating GR activity over NF-κB activation. This effect may be accomplished by means of GR parylation or physical interaction between these two molecules. These alternatives remain to be explored.

## Conclusion

The neuroendocrine system has a determinant role in the control of inflammatory mechanisms, in order to allow the organism to return to homeostasis and therefore avoid pathological situations of exacerbated inflammation. In this context, both GR and PARP1 have prominent antagonic roles in the regulation of inflammatory processes. Although PARP1 and NRs have been reported to functionally interact, there have not been reports so far showing interaction between PARP and GR. It would be of interest to address this issue, in order to confirm either a direct or indirect interaction as it is the case between PARP1 and other NRs, where PARP1 is a component of the transcriptional complex that mediates steroid-driven transcription. Considering that PARP1 and GR do share common targets involved in inflammatory responses, the possibility that PARP1 may have a role in the regulation of cytokine and other inflammatory mediators expression mediated by GCs at the CNS level arises. In this context, it would also be interesting to explore whether through the mechanisms discussed above PARP1 may be playing a role in mediating the well-known patient GC resistance in inflammatory disease. The understanding of the molecular mechanism leading to the antagonic effect of these two regulators may provide novel targets in the neuroendocrine control of inflammation.

## References

[1] Medzhitov R (2008). Origin and physiological roles of inflammation. Nature.

[2] Dinarello CA (2010). Anti-inflammatory agents: present and future. Cell.

[3] Chiu R, Boyle WJ, Meek J, Smeal T, Hunter T, Karin M (1988). The c-Fos protein interacts with c-Jun/AP-1 to stimulate transcription of AP-1 responsive genes. Cell.

[4] Auphan N, DiDonato JA, Rosette C, Helmberg A, Karin M (1995). Immunosuppression by glucocorticoids: inhibition of NF-κB activity through induction of IκB synthesis. Science.

[5] Hayden MS, Ghosh S (2008). Shared principles in NF-κB signaling. Cell.

[6] Elenkov IJ, Iezzoni DG, Daly A, Harris AG, Chrousos GP (2005). Cytokine dysregulation, inflammation and well-being. Neuroimmunomodulation.

[7] Ridker PM, Hennekens CH, Roitman-Johnson B, Stampfer MJ, Allen J (1998). Plasma concentration of soluble intercellular adhesion molecule 1 and risks of future myocardial infarction in apparently healthy men. Lancet.

[8] Rohleder N (2012). Acute and chronic stress induced changes in sensitivity of peripheral inflammatory pathways to the signals of multiple stress systems – 2011 Curt Richter Award Winner. Psychoneuroendocrinology.

[9] Dantzer R, O'Connor JC, Freund GG, Johnson RW, Kelley KW (2008). From inflammation to sickness and depression: when the immune system subjugates the brain. Nature Reviews. Neuroscience.

[10] Besedovsky HO, del Rey A (1992). Immune–neuroendocrine circuits: integrative role of cytokines. Frontiers in Neuroendocrinology.

[11] De Kloet ER, Vreugdenhil E, Oitzl MS, Joels M (1998). Brain corticosteroid receptor balance in health and disease. Endocrine Reviews.

[12] Smith SM, Vale WW (2006). The role of the hypothalamic–pituitary–adrenal axis in neuroendocrine responses to stress. Dialogues in Clinical Neuroscience.

[13] Green PG, Miao FJ, Janig W, Levine JD (1995). Negative feedback neuroendocrine control of the inflammatory response in rats. Journal of Neuroscience.

[14] McKay LI, Cidlowski JA (1999). Molecular control of immune/inflammatory responses: interactions between nuclear factor-κB and steroid receptor-signaling pathways. Endocrine Reviews.

[15] Besedovsky H, del Rey A, Sorkin E, Dinarello CA (1986). Immunoregulatory feedback between interleukin-1 and glucocorticoid hormones. Science.

[16] de Kloet ER, Joels M, Holsboer F (2005). Stress and the brain: from adaptation to disease. Nature Reviews. Neuroscience.

[17] Arzt E, Pereda MP, Castro CP, Pagotto U, Renner U, Stalla GK (1999). Pathophysiological role of the cytokine network in the anterior pituitary gland. Frontiers in Neuroendocrinology.

[18] Liberman AC, Druker J, Perone MJ, Arzt E (2007). Glucocorticoids in the regulation of transcription factors that control cytokine synthesis. Cytokine & Growth Factor Reviews.

[19] Sapolsky RM, Romero LM, Munck AU (2000). How do glucocorticoids influence stress responses? Integrating permissive, suppressive, stimulatory, and preparative actions. Endocrine Reviews.

[20] Cooper MS, Bujalska I, Rabbitt E, Walker EA, Bland R, Sheppard MC, Hewison M, Stewart PM (2001). Modulation of 11β-hydroxysteroid dehydrogenase isozymes by proinflammatory cytokines in osteoblasts: an autocrine switch from glucocorticoid inactivation to activation. Journal of Bone and Mineral Research.

[21] Hardy RS, Filer A, Cooper MS, Parsonage G, Raza K, Hardie DL, Rabbitt EH, Stewart PM, Buckley CD, Hewison M (2006). Differential expression, function and response to inflammatory stimuli of 11β-hydroxysteroid dehydrogenase type 1 in human fibroblasts: a mechanism for tissue-specific regulation of inflammation. Arthritis Research & Therapy.

[22] Kaur K, Hardy R, Ahasan MM, Eijken M, van Leeuwen JP, Filer A, Thomas AM, Raza K, Buckley CD, Stewart PM (2010). Synergistic induction of local glucocorticoid generation by inflammatory cytokines and glucocorticoids: implications for inflammation associated bone loss. Annals of the Rheumatic Diseases.

[23] Ahasan MM, Hardy R, Jones C, Kaur K, Nanus D, Juarez M, Morgan SA, Hassan-Smith Z, Benezech C, Caamano JH (2012). Inflammatory regulation of glucocorticoid metabolism in mesenchymal stromal cells. Arthritis and Rheumatism.

[24] Raza K, Hardy R, Cooper MS (2010). The 11β-hydroxysteroid dehydrogenase enzymes – arbiters of the effects of glucocorticoids in synovium and bone. Rheumatology.

[25] Beck IM, Vanden Berghe W, Vermeulen L, Yamamoto KR, Haegeman G, De Bosscher K (2009). Crosstalk in inflammation: the interplay of glucocorticoid receptor-based mechanisms and kinases and phosphatases. Endocrine Reviews.

[26] Schaaf MJ, Lewis-Tuffin LJ, Cidlowski JA (2005). Ligand-selective targeting of the glucocorticoid receptor to nuclear subdomains is associated with decreased receptor mobility. Molecular Endocrinology.

[27] Vandevyver S, Dejager L, Libert C (2012). On the trail of the glucocorticoid receptor: into the nucleus and back. Traffic.

[28] Clark AR (2007). Anti-inflammatory functions of glucocorticoid-induced genes. Molecular and Cellular Endocrinology.

[29] De Bosscher K, Vanden Berghe W, Haegeman G (2003). The interplay between the glucocorticoid receptor and nuclear factor-κB or activator protein-1: molecular mechanisms for gene repression. Endocrine Reviews.

[30] Rhen T, Cidlowski JA (2005). Antiinflammatory action of glucocorticoids – new mechanisms for old drugs. New England Journal of Medicine.

[31] Scheinman RI, Gualberto A, Jewell CM, Cidlowski JA, Baldwin AS (1995). Characterization of mechanisms involved in transrepression of NF-κB by activated glucocorticoid receptors. Molecular and Cellular Biology.

[32] Kassel O, Herrlich P (2007). Crosstalk between the glucocorticoid receptor and other transcription factors: molecular aspects. Molecular and Cellular Endocrinology.

[33] Pascual G, Glass CK (2006). Nuclear receptors versus inflammation: mechanisms of transrepression. Trends in Endocrinology and Metabolism.

[34] De Bosscher K, Vanden Berghe W, Haegeman G (2006). Cross-talk between nuclear receptors and nuclear factor κB. Oncogene.

[35] Jonat C, Rahmsdorf HJ, Park KK, Cato AC, Gebel S, Ponta H, Herrlich P (1990). Antitumor promotion and antiinflammation: down-modulation of AP-1 (Fos/Jun) activity by glucocorticoid hormone. Cell.

[36] Helmberg A, Auphan N, Caelles C, Karin M (1995). Glucocorticoid-induced apoptosis of human leukemic cells is caused by the repressive function of the glucocorticoid receptor. EMBO Journal.

[37] Kovalovsky D, Refojo D, Holsboer F, Arzt E (2000). Molecular mechanisms and Th1/Th2 pathways in corticosteroid regulation of cytokine production. Journal of Neuroimmunology.

[38] Vacca A, Felli MP, Farina AR, Martinotti S, Maroder M, Screpanti I, Meco D, Petrangeli E, Frati L, Gulino A (1992). Glucocorticoid receptor-mediated suppression of the interleukin 2 gene expression through impairment of the cooperativity between nuclear factor of activated T cells and AP-1 enhancer elements. Journal of Experimental Medicine.

[39] De Bosscher K, Schmitz ML, Vanden Berghe W, Plaisance S, Fiers W, Haegeman G (1997). Glucocorticoid-mediated repression of nuclear factor-κB-dependent transcription involves direct interference with transactivation. PNAS.

[40] De Bosscher K, Haegeman G (2009). Minireview: latest perspectives on antiinflammatory actions of glucocorticoids. Molecular Endocrinology.

[41] Liberman AC, Druker J, Refojo D, Holsboer F, Arzt E (2009). Glucocorticoids inhibit GATA-3 phosphorylation and activity in T cells. FASEB Journal.

[42] Liberman AC, Refojo D, Druker J, Toscano M, Rein T, Holsboer F, Arzt E (2007). The activated glucocorticoid receptor inhibits the transcription factor T-bet by direct protein–protein interaction. FASEB Journal.

[43] Besedovsky HO, del Rey A (2006). Regulating inflammation by glucocorticoids. Nature Immunology.

[44] Liberman AC, Castro CN, Antunica Noguerol M, Barcala Tabarrozzi AE, Druker J, Perone MJ, Arzt E (2010). Molecular mechanisms of glucocorticoids action: from basic research to clinical implications. Current Immunology Reviews.

[45] De Bosscher K, Beck IM, Haegeman G (2010). Classic glucocorticoids versus non-steroidal glucocorticoid receptor modulators: survival of the fittest regulator of the immune system?. Brain, Behavior, and Immunity.

[46] De Bosscher K, Haegeman G, Elewaut D (2010). Targeting inflammation using selective glucocorticoid receptor modulators. Current Opinion in Pharmacology.

[47] De Bosscher K, Van Craenenbroeck K, Meijer OC, Haegeman G (2008). Selective transrepression versus transactivation mechanisms by glucocorticoid receptor modulators in stress and immune systems. European Journal of Pharmacology.

[48] Belvisi MG, Wicks SL, Battram CH, Bottoms SE, Redford JE, Woodman P, Brown TJ, Webber SE, Foster ML (2001). Therapeutic benefit of a dissociated glucocorticoid and the relevance of *in vitro* separation of transrepression from transactivation activity. Journal of Immunology.

[49] Vayssiere BM, Dupont S, Choquart A, Petit F, Garcia T, Marchandeau C, Gronemeyer H, Resche-Rigon M (1997). Synthetic glucocorticoids that dissociate transactivation and AP-1 transrepression exhibit antiinflammatory activity *in vivo*. Molecular Endocrinology.

[50] De Bosscher K, Vanden Berghe W, Beck IM, Van Molle W, Hennuyer N, Hapgood J, Libert C, Staels B, Louw A, Haegeman G (2005). A fully dissociated compound of plant origin for inflammatory gene repression. PNAS.

[51] De Bosscher K, Beck IM, Dejager L, Bougarne N, Gaigneaux A, Chateauvieux S, Ratman D, Bracke M, Tavernier J, Vanden Berghe W (2013). Selective modulation of the glucocorticoid receptor can distinguish between transrepression of NF-κB and AP-1. Cellular and Molecular Life Sciences.

[52] So AY, Chaivorapol C, Bolton EC, Li H, Yamamoto KR (2007). Determinants of cell- and gene-specific transcriptional regulation by the glucocorticoid receptor. PLoS Genetics.

[53] Barnes PJ (2011). Glucocorticosteroids: current and future directions. British Journal of Pharmacology.

[54] Mangelsdorf DJ, Thummel C, Beato M, Herrlich P, Schutz G, Umesono K, Blumberg B, Kastner P, Mark M, Chambon P (1995). The nuclear receptor superfamily: the second decade. Cell.

[55] Dijsselbloem N, Vanden Berghe W, De Naeyer A, Haegeman G (2004). Soy isoflavone phyto-pharmaceuticals in interleukin-6 affections. Multi-purpose nutraceuticals at the crossroad of hormone replacement, anti-cancer and anti-inflammatory therapy. Biochemical Pharmacology.

[56] Cinar B, De Benedetti A, Freeman MR (2005). Post-transcriptional regulation of the androgen receptor by mammalian target of rapamycin. Cancer Research.

[57] Mauro C, Zazzeroni F, Papa S, Bubici C, Franzoso G (2009). The NF-κB transcription factor pathway as a therapeutic target in cancer: methods for detection of NF-κB activity. Methods in Molecular Biology.

[58] Gonzalez-Angulo AM, Morales-Vasquez F, Hortobagyi GN (2007). Overview of resistance to systemic therapy in patients with breast cancer. Advances in Experimental Medicine and Biology.

[59] Hermanson O, Glass CK, Rosenfeld MG (2002). Nuclear receptor coregulators: multiple modes of modification. Trends in Endocrinology and Metabolism.

[60] Kim MY, Woo EM, Chong YT, Homenko DR, Kraus WL (2006). Acetylation of estrogen receptor α by p300 at lysines 266 and 268 enhances the deoxyribonucleic acid binding and transactivation activities of the receptor. Molecular Endocrinology.

[61] Perkins ND (2006). Post-translational modifications regulating the activity and function of the nuclear factor κB pathway. Oncogene.

[62] Ito K, Yamamura S, Essilfie-Quaye S, Cosio B, Ito M, Barnes PJ, Adcock IM (2006). Histone deacetylase 2-mediated deacetylation of the glucocorticoid receptor enables NF-κB suppression. Journal of Experimental Medicine.

[63] Ame JC, Spenlehauer C, de Murcia G (2004). The PARP superfamily. BioEssays.

[64] Otto H, Reche PA, Bazan F, Dittmar K, Haag F, Koch-Nolte F (2005). *In silico* characterization of the family of PARP-like poly(ADP-ribosyl)transferases (pARTs). BMC Genomics.

[65] D'Amours D, Desnoyers S, D'Silva I, Poirier GG (1999). Poly(ADP-ribosyl)ation reactions in the regulation of nuclear functions. Biochemical Journal.

[66] Zhang Y, Wang J, Ding M, Yu Y (2013). Site-specific characterization of the Asp- and Glu-ADP-ribosylated proteome. Nature Methods.

[67] Altmeyer M, Messner S, Hassa PO, Fey M, Hottiger MO (2009). Molecular mechanism of poly(ADP-ribosyl)ation by PARP1 and identification of lysine residues as ADP-ribose acceptor sites. Nucleic Acids Research.

[68] Hassa PO, Hottiger MO (2008). The diverse biological roles of mammalian PARPS, a small but powerful family of poly-ADP-ribose polymerases. Frontiers in Bioscience.

[69] Benjamin RC, Gill DM (1980). Poly(ADP-ribose) synthesis *in vitro* programmed by damaged DNA. A comparison of DNA molecules containing different types of strand breaks. Journal of Biological Chemistry.

[70] Kraus WL, Hottiger MO (2013). PARP-1 and gene regulation: progress and puzzles. Molecular Aspects of Medicine.

[71] Kraus WL, Lis JT (2003). PARP goes transcription. Cell.

[72] Mendoza-Alvarez H, Alvarez-Gonzalez R (2001). Regulation of p53 sequence-specific DNA-binding by covalent poly(ADP-ribosyl)ation. Journal of Biological Chemistry.

[73] Kraus WL (2008). Transcriptional control by PARP-1: chromatin modulation, enhancer-binding, coregulation, and insulation. Current Opinion in Cell Biology.

[74] Ji Y, Tulin AV (2010). The roles of PARP1 in gene control and cell differentiation. Current Opinion in Genetics & Development.

[75] Krishnakumar R, Kraus WL (2010). The PARP side of the nucleus: molecular actions, physiological outcomes, and clinical targets. Molecular Cell.

[76] Huletsky A, de Murcia G, Muller S, Hengartner M, Menard L, Lamarre D, Poirier GG (1989). The effect of poly(ADP-ribosyl)ation on native and H1-depleted chromatin. A role of poly(ADP-ribosyl)ation on core nucleosome structure. Journal of Biological Chemistry.

[77] Poirier GG, de Murcia G, Jongstra-Bilen J, Niedergang C, Mandel P (1982). Poly(ADP-ribosyl)ation of polynucleosomes causes relaxation of chromatin structure. PNAS.

[78] Kim MY, Mauro S, Gevry N, Lis JT, Kraus WL (2004). NAD+-dependent modulation of chromatin structure and transcription by nucleosome binding properties of PARP-1. Cell.

[79] Wacker DA, Ruhl DD, Balagamwala EH, Hope KM, Zhang T, Kraus WL (2007). The DNA binding and catalytic domains of poly(ADP-ribose) polymerase 1 cooperate in the regulation of chromatin structure and transcription. Molecular and Cellular Biology.

[80] Krishnakumar R, Gamble MJ, Frizzell KM, Berrocal JG, Kininis M, Kraus WL (2008). Reciprocal binding of PARP-1 and histone H1 at promoters specifies transcriptional outcomes. Science.

[81] Ju BG, Lunyak VV, Perissi V, Garcia-Bassets I, Rose DW, Glass CK, Rosenfeld MG (2006). A topoisomerase IIβ-mediated dsDNA break required for regulated transcription. Science.

[82] Slattery E, Dignam JD, Matsui T, Roeder RG (1983). Purification and analysis of a factor which suppresses nick-induced transcription by RNA polymerase II and its identity with poly(ADP-ribose) polymerase. Journal of Biological Chemistry.

[83] Hassa PO, Buerki C, Lombardi C, Imhof R, Hottiger MO (2003). Transcriptional coactivation of nuclear factor-κB-dependent gene expression by p300 is regulated by poly(ADP)-ribose polymerase-1. Journal of Biological Chemistry.

[84] Hassa PO, Haenni SS, Buerki C, Meier NI, Lane WS, Owen H, Gersbach M, Imhof R, Hottiger MO (2005). Acetylation of poly(ADP-ribose) polymerase-1 by p300/CREB-binding protein regulates coactivation of NF-κB-dependent transcription. Journal of Biological Chemistry.

[85] Pavri R, Lewis B, Kim TK, Dilworth FJ, Erdjument-Bromage H, Tempst P, de Murcia G, Evans R, Chambon P, Reinberg D (2005). PARP-1 determines specificity in a retinoid signaling pathway via direct modulation of mediator. Molecular Cell.

[86] Attwood JT, Yung RL, Richardson BC (2002). DNA methylation and the regulation of gene transcription. Cellular and Molecular Life Sciences.

[87] Caiafa P, Zampieri M (2005). DNA methylation and chromatin structure: the puzzling CpG islands. Journal of Cellular Biochemistry.

[88] Caiafa P, Guastafierro T, Zampieri M (2009). Epigenetics: poly(ADP-ribosyl)ation of PARP-1 regulates genomic methylation patterns. FASEB Journal.

[89] Reale A, Matteis GD, Galleazzi G, Zampieri M, Caiafa P (2005). Modulation of DNMT1 activity by ADP-ribose polymers. Oncogene.

[90] Schiewer MJ, Goodwin JF, Han S, Brenner JC, Augello MA, Dean JL, Liu F, Planck JL, Ravindranathan P, Chinnaiyan AM (2012). Dual roles of PARP-1 promote cancer growth and progression. Cancer Discovery.

[91] Sartorius CA, Takimoto GS, Richer JK, Tung L, Horwitz KB (2000). Association of the Ku autoantigen/DNA-dependent protein kinase holoenzyme and poly(ADP-ribose) polymerase with the DNA binding domain of progesterone receptors. Journal of Molecular Endocrinology.

[92] Wright RH, Castellano G, Bonet J, Le Dily F, Font-Mateu J, Ballare C, Nacht AS, Soronellas D, Oliva B, Beato M (2012). CDK2-dependent activation of PARP-1 is required for hormonal gene regulation in breast cancer cells. Genes and Development.

[93] Inbar-Rozensal D, Castiel A, Visochek L, Castel D, Dantzer F, Izraeli S, Cohen-Armon M (2009). A selective eradication of human nonhereditary breast cancer cells by phenanthridine-derived polyADP-ribose polymerase inhibitors. Breast Cancer Research.

[94] Rehman FL, Lord CJ, Ashworth A (2012). The promise of combining inhibition of PI3K and PARP as cancer therapy. Cancer Discovery.

[95] Cuzzocrea S (2005). Shock, inflammation and PARP. Pharmacological Research.

[96] Nossa CW, Jain P, Tamilselvam B, Gupta VR, Chen LF, Schreiber V, Desnoyers S, Blanke SR (2009). Activation of the abundant nuclear factor poly(ADP-ribose) polymerase-1 by *Helicobacter pylori*. PNAS.

[97] Ha HC (2004). Defective transcription factor activation for proinflammatory gene expression in poly(ADP-ribose) polymerase 1-deficient glia. PNAS.

[98] Mabley JG, Suarez-Pinzon WL, Hasko G, Salzman AL, Rabinovitch A, Kun E, Szabo C (2001). Inhibition of poly (ADP-ribose) synthetase by gene disruption or inhibition with 5-iodo-6-amino-1,2-benzopyrone protects mice from multiple-low-dose-streptozotocin-induced diabetes. British Journal of Pharmacology.

[99] Chiarugi A, Moskowitz MA (2003). Poly(ADP-ribose) polymerase-1 activity promotes NF-κB-driven transcription and microglial activation: implication for neurodegenerative disorders. Journal of Neurochemistry.

[100] Ha HC, Hester LD, Snyder SH (2002). Poly(ADP-ribose) polymerase-1 dependence of stress-induced transcription factors and associated gene expression in glia. PNAS.

[101] Lee JH, Park SY, Shin HK, Kim CD, Lee WS, Hong KW (2007). Poly(ADP-ribose) polymerase inhibition by cilostazol is implicated in the neuroprotective effect against focal cerebral ischemic infarct in rat. Brain Research.

[102] Moroni F (2008). Poly(ADP-ribose)polymerase 1 (PARP-1) and postischemic brain damage. Current Opinion in Pharmacology.

[103] Nakajima H, Nagaso H, Kakui N, Ishikawa M, Hiranuma T, Hoshiko S (2004). Critical role of the automodification of poly(ADP-ribose) polymerase-1 in nuclear factor-κB-dependent gene expression in primary cultured mouse glial cells. Journal of Biological Chemistry.

[104] Weltin D, Picard V, Aupeix K, Varin M, Oth D, Marchal J, Dufour P, Bischoff P (1995). Immunosuppressive activities of 6(5H)-phenanthridinone, a new poly(ADP-ribose)polymerase inhibitor. International Journal of Immunopharmacology.

[105] Hassa PO, Hottiger MO (1999). A role of poly (ADP-ribose) polymerase in NF-κB transcriptional activation. Biological Chemistry.

[106] Oliver FJ, Menissier-de Murcia J, Nacci C, Decker P, Andriantsitohaina R, Muller S, de la Rubia G, Stoclet JC, de Murcia G (1999). Resistance to endotoxic shock as a consequence of defective NF-κB activation in poly (ADP-ribose) polymerase-1 deficient mice. EMBO Journal.

[107] Zingarelli B, Hake PW, Burroughs TJ, Piraino G, O'Connor M, Denenberg A (2004). Activator protein-1 signalling pathway and apoptosis are modulated by poly(ADP-ribose) polymerase-1 in experimental colitis. Immunology.

[108] Olabisi OA, Soto-Nieves N, Nieves E, Yang TT, Yang X, Yu RY, Suk HY, Macian F, Chow CW (2008). Regulation of transcription factor NFAT by ADP-ribosylation. Molecular and Cellular Biology.

[109] Valdor R, Schreiber V, Saenz L, Martinez T, Munoz-Suano A, Dominguez-Villar M, Ramirez P, Parrilla P, Aguado E, Garcia-Cozar F (2008). Regulation of NFAT by poly(ADP-ribose) polymerase activity in T cells. Molecular Immunology.

[110] Bai P, Canto C, Oudart H, Brunyanszki A, Cen Y, Thomas C, Yamamoto H, Huber A, Kiss B, Houtkooper RH (2011). PARP-1 inhibition increases mitochondrial metabolism through SIRT1 activation. Cell Metabolism.

[111] Kauppinen TM, Swanson RA (2007). The role of poly(ADP-ribose) polymerase-1 in CNS disease. Neuroscience.

[112] Eliasson MJ, Sampei K, Mandir AS, Hurn PD, Traystman RJ, Bao J, Pieper A, Wang ZQ, Dawson TM, Snyder SH (1997). Poly(ADP-ribose) polymerase gene disruption renders mice resistant to cerebral ischemia. Nature Medicine.

[113] Ros-Bernal F, Hunot S, Herrero MT, Parnadeau S, Corvol JC, Lu L, Alvarez-Fischer D, Carrillo-de Sauvage MA, Saurini F, Coussieu C (2011). Microglial glucocorticoid receptors play a pivotal role in regulating dopaminergic neurodegeneration in parkinsonism. PNAS.

[114] Bhatt AJ, Feng Y, Wang J, Famuyide M, Hersey K (2013). Dexamethasone induces apoptosis of progenitor cells in the subventricular zone and dentate gyrus of developing rat brain. Journal of Neuroscience Research.

[115] Alano CC, Garnier P, Ying W, Higashi Y, Kauppinen TM, Swanson RA (2010). NAD+ depletion is necessary and sufficient for poly(ADP-ribose) polymerase-1-mediated neuronal death. Journal of Neuroscience.

[116] Tang KS, Suh SW, Alano CC, Shao Z, Hunt WT, Swanson RA, Anderson CM (2010). Astrocytic poly(ADP-ribose) polymerase-1 activation leads to bioenergetic depletion and inhibition of glutamate uptake capacity. Glia.

[117] Scott GS, Hake P, Kean RB, Virag L, Szabo C, Hooper DC (2001). Role of poly(ADP-ribose) synthetase activation in the development of experimental allergic encephalomyelitis. Journal of Neuroimmunology.

[118] Love S, Barber R, Wilcock GK (1999). Increased poly(ADP-ribosyl)ation of nuclear proteins in Alzheimer's disease. Brain.

[119] Abeti R, Abramov AY, Duchen MR (2011). β-Amyloid activates PARP causing astrocytic metabolic failure and neuronal death. Brain.

